# Diagnosis and management of multiple primary lung cancer

**DOI:** 10.3389/fonc.2024.1392969

**Published:** 2024-10-01

**Authors:** Honghong Dong, Yahui Tian, Shaowei Xin, Suxin Jiang, Yujie Guo, Zitong Wan, Yong Han

**Affiliations:** ^1^ Department of Thoracic Surgery, Air Force Medical Center, Air Force Medical University, Beijing, China; ^2^ Department of Thoracic Surgery, 962 Hospital of the joint Logistics Support Force, Harbin, China; ^3^ Graduate School of China Medical University, Shenyang, China; ^4^ College of Life Sciences, Northwestern University, Xi’an, China

**Keywords:** multiple primary lung cancer, diagnosis, management, surgery, histology

## Abstract

Multiple primary lung cancer (MPLC), can be categorized as synchronous multiple primary lung cancer (sMPLC) and metachronous multiple primary lung cancer (mMPLC), which are becoming increasingly common in clinical practice. A precise differential diagnosis between MPLC and intrapulmonary metastases (IPM) is essential for determining the appropriate management strategy. MPLC is primarily diagnosed through histology, imaging, and molecular methods. Imaging serves as an essential foundation for preoperative diagnosis, while histology is a critical tool for establishing a definitive diagnosis. As molecular biology advances, the diagnosis of MPLC has stepped into the era of molecular precision. Surgery is the preferred treatment approach, with stereotactic radiotherapy and ablation being viable options for unresectable lesions. Targeted therapy and immunotherapy can be considered for specific patients. A multidisciplinary team approach to evaluation and the application of combination therapy can benefit more patients. Looking ahead, the development of more authoritative guidelines will be instrumental in streamlining the diagnosis and management of MPLC.

## 1 Introduction

Multiple primary lung cancer (MPLC) refers to the presence of multiple primary lung cancer lesions in the lung, which can be classified as synchronous multiple primary lung cancer (sMPLC) and metachronous multiple primary lung cancer (mMPLC) according to the time of lesion occurrence. In recent years, with the widespread use of low-dose computed tomography (LDCT), an increasing number of MPLCs have been diagnosed, attracting the attention of clinicians. The differentiation between MPLC and intrapulmonary metastasis (IPM) is particularly important, as different diagnoses imply completely different stages and treatment plans. However, the complexity and uncertainty of the onset of MPLC make its diagnosis and management a problem that troubles clinical doctors.

Diagnostic criteria for MPLC is updated over time. Martini and Melamed (M-M) (1) established the earliest histologic diagnostic criteria in 1975, which laid the foundation for the diagnosis of MPLC (2, 3). Up to now, several organizations have published their own diagnostic criteria (2, 3). However, there is still a lack of universal and authoritative diagnostic criteria for standardized clinical practice. With rapid development in the field of molecular biology research, the diagnosis of MPLC has also entered the era of molecular diagnosis. Therefore, the diagnosis of MPLC is in dire need of an authoritative update.

According to the latest NCCN guidelines (version 5.2024), the treatment of MPLC should be customized by a multidisciplinary team, including thoracic radiology, pulmonary medicine, thoracic surgery, medical oncology, and radiation oncology. Surgery is the mainstay of MPLC treatment and the choice of surgical modality often confuses clinicians. Whether to perform simultaneous resections of multiple foci or multiple surgical resections, and whether to operate on *de novo* foci, etc. Stereotactic body radiation therapy and image guided thermal ablation are currently the treatment for inoperable MPLC, with favorable outcomes. The use of targeted therapy and immunotherapy in patients with MPLC is still in the exploratory stage, and long-term prognostic information is not yet available. Treatment options for MPLC still need to be further standardized.

In this review, we will focus on advances in the diagnosis and management of MPLC to provide a strong reference for the clinical diagnosis and management of MPLC.

## 2 Diagnosis

### 2.1 Histopathological diagnosis

#### 2.1.1 1975 M-M

In 1975, Martini and Melamed (1) first proposed histological diagnostic criteria for MPLC based on information from 50 patients with multiple primary lung cancer. M-M is the first proposed diagnostic standard for MPLC and is therefore widely cited. However, its establishment mainly relied on the diagnostic experience of clinicians at that time, which was a relatively rough diagnostic criterion. Subsequent research (6) found that the M-M standard has significant limitations in identifying IPM and has a poor ability to distinguish prognosis (7).

#### 2.1.2 2007/2013 ACCP guidelines

The 2^nd^ clinical practice guidelines released by the ACCP in 2007 proposed new diagnostic criteria for MPLC, stating that the diagnostic interval for mMPLC should be ≥ 4 years (3). The ACCP guidelines indicate that a time interval of less than 2 years is more likely to be associated with pulmonary metastasis, and the incidence of new primary tumors and pulmonary metastasis within 2-4 years is equivalent. When the time interval is ≥ 4 years or more, the lesion should be considered a primary tumor. The 3^rd^ edition published in 2013 (8) followed the content of the 2^nd^ edition without significant differences. A meta-analysis reviewing 22 studies between 1975 and 2013 found that the median tumor-free interval of mMPLC (n=883) was 24-71 m. It is evident that there is uncertainty in the timing of the second primary lung cancer (SPLC) in mMPLC. A rigid time standard poses the risk of missed diagnosis and misdiagnosis.

#### 2.1.3 2017 AJCC

The 8th TNM staging published by AJCC in 2017 proposed the use of comprehensive histological assessment (CHA) to distinguish sMPLC from IPM (9), and a separate staging was performed for MPLC (2). CHA distinguishes between multiple primary and intrapulmonary metastases by sequentially comparing the histologic types, tissue subtypes, and cytologic and mesenchymal features and has shown good diagnostic performance among pathological experts; the consistency with molecular evaluation results can reach 91% (10). However, CHA is based on a limited number of cancer patients and is not the so-called gold standard. Studies (11–16) have shown that the histologic diagnosis is unclear in approximately 15-29.8% of patients, especially IPM, which is prone to misdiagnosis.

There is other pathologic feature can predict prognosis of MPLC patients. A recent retrospective study showed that spread through air spaces (STAS) has an impact on the postoperative recurrence-free survival of patients with MPLC. STAS (+) had a significantly higher recurrence rate than STAS (–) (34% vs. 0%, p<0.05) (17).

As of today, there are no recognized diagnostic criteria for MPLC. Histology is the gold standard for cancer diagnosis, but there is still a risk of misdiagnosis for some MPLC. Discovery of new pathologic features associated with MPLC is needed to clarify the pathologic diagnosis and prognosis of MPLC.

### 2.2 Molecular diagnosis

With the rapid development of molecular biology research and the continuous upgrading and iteration of sequencing technology, the diagnosis of MPLC has moved into the era of molecular diagnosis. Researchers began to test genetic features to explain whether there is a clonal relationship between multiple lesions. The ACCP 2^nd^ guidelines first proposed the inclusion of molecular genetic features in the diagnostic criteria in 2007 (3). It is generally believed that the primary and metastatic lesions originate from the same clone, so there are shared molecular features between the two, while there is no significant correlation between multiple primary lesions. Subsequent studies have confirmed that different primary lesions in the same patient have different genomic characteristics (18) and a 96% concordance between driving mutations in primary and metastatic lesions (11). Despite heterogeneity within tumors, approximately 70-76% of mutations are shared across all sites, and a single biopsy analysis at an appropriate depth may be sufficient to identify most known cancer-related gene mutations in lung cancer (19, 20).

In previous studies, scholars have attempted to distinguish MPLC and IPM by detecting single nucleotide polymorphisms (SNPs), microsatellite instability (MSI) (21, 22), cancer-related gene mutations (6, 7, 11–16, 18, 23–36), copy number variations (CNVs) (18, 24, 29, 37) loss of heterozygosity (LOH) (38), chromosomal rearrangements (13, 18, 29, 39, 40), and chromosomal inactivation.

#### 2.2.1 Cancer-related gene mutation detection

Researchers first began to detect mutations in the oncogene P53 in tumor tissue from lung cancer patients with two lesions (27, 28), which occurred before metastasis and were conserved during metastasis. Most (95-98%) are found in exons 5-9. P53 mutations were detected in approximately 35% to 56% of participants, and there were three mutation patterns: A) mutations in only one tumor, B) different P53 mutations in two tumors, and C) the same P53 mutation in two tumors. The first two mutation patterns are considered MPLC, and the last one is IPM.

In addition, more studies began to detect multiple cancer gene mutations in lesions for molecular diagnosis (**Table 1**). In the study by Asmar et al., 45 patients with primary tumors and metastases (cohort 1) and 69 patients with multiple tumors (cohort 2, 154 tumors) were included, and their samples were tested for *EGFR, KRAS, ALK*, and *BRAF* gene mutations (11). Sharing driver mutations between multiple tumors was considered metastasis and multiple primary tumors if different driver mutations were present. The results showed a 96% compliance rate of driver gene mutations between primary tumors and metastases in cohort 1 and a 29.8% discordance rate between molecular and histological diagnoses in cohort 2. Zheng et al. showed that a 4-gene (*EGFR, KRAS, BRAF*, and *NRAS*) next-generation sequencing (NGS) panel better differentiated MPLC and IPM, while conventional histology misdiagnosed 4 groups of lesions (4/18, 22.2%) (16). Goodwin et al. examined mutations in more than 50 genes (*ALK, BRAF, EGFR, KRAS, MET, TP53*, etc.) in patients with double primary lung cancer (n=40), which resulted in a match with the clinicopathological diagnosis in 82.5% (33/40) of patients (13). In the study by Mansuet-Lupo, mutations in 22 genes (*KRAS, EGFR, BRAF, TP53*, etc.) were examined in patients with multiple lung adenocarcinomas (n=120), and the results showed that mutations were detected in 91% of the patients (14). Twenty-seven percent of cases (30/109) were inconsistent with the histologic diagnosis. In addition, 341-468 genes and somatic mutations and structural variants were detected in patients with multiple lung cancers using (MSK-IMPACT) NGS in Chang’s study (12). Zhanget al. used the NGS panel to detect 425 tumor-related genes and found that approximately 15-17% of cases did not match the results of the CHA (15). At present, there are no results on the optimal number of genes to be detected, and some studies suggest that a panel needs at least 100 genes to confirm the cloning relationship of 95% of adenocarcinoma (41). Of note, if multiple lesions share only one hotspot mutation, such as EGFR p.L858R (which occurs in approximately 40% of Asian lung adenocarcinoma patients (42)), the presence of other shared somatic mutations should be cautiously reviewed to determine the clonal relationship between multiple tumors (12, 18).

**Table 1:** Histopathological diagnosis of multiple primary lung cancer.

Diagnosis criteria	References
**1975 M-M**	(1)
Metachronous tumors	
A. Histology different	
B. Histology the same, if:	
I. Free interval between cancers at least 2 years or	
2. Origin from carcinoma *in situ* or	
3. Second cancer in different lobe or lung, but:	
a. No carcinoma in lymphatics common to both	
b. No extrapulmonary metastases at time of diagnosis	
Synchronous tumors	
A. Tumors physically distinct and separate	
B. Histology:	
I. Different	
2. Same, but in different segment, lobe, or lung, if:	
a. Origin from carcinoma in situ	
b. No carcinoma in lymphatics common to both	
c. No extrapulmonary metastases at time of diagnosis	
**2007/2013 ACCP**	(3, 8)
Same histology, anatomically separated	
Cancers in different lobes	
And no N2,3 involvement	
And no systemic metastases	
Same histology, temporally separated	
≥ 4-yr interval between cancers	
And no systemic metastases from either cancer	
Different histology	
Different histologic type	
Or different molecular genetic characteristics	
Or arising separately from foci of carcinoma in situ	
Hematogenously spread pulmonary metastases	
Same histology and multiple systemic metastases	
Same histology, in different lobes	
And presence of N2,3 involvement	
Or < 2-yr interval	
**2017 AJCC**	(9)
Clinical criteria	
Tumors may be considered separate primary tumors if:	
They are clearly of a different histologic type (eg, squamous carcinoma and adenocarcinoma) by biopsy	
Tumors may be considered to be arising from a single tumor source if:	
Exactly matching breakpoints are identified by comparative genomic hybridization	
Relative arguments that favor separate tumors:	
Different radiographic appearance or metabolic uptake	
Different biomarker pattern (driver gene mutations)	
Different rates of growth (if previous imaging is available)	
Absence of nodal or systemic metastases	
Relative arguments that favor a single tumor source:	
Same radiographic appearance	
Similar growth patterns (if previous imaging is available)	
Significant nodal or systemic metastases	
Same biomarker pattern (and same histotype)	
Pathologic criteria (ie, after resection)	
Tumors may be considered separate primary tumors if:	
They are clearly of a different histologic type (eg, squamous carcinoma and adenocarcinoma)	
They are clearly different by a comprehensive histologic assessment	
They are squamous carcinomas that have arisen from carcinoma in situ	
Tumors may be considered to be arising from a single tumor source if:	
Exactly matching breakpoints are identified by comparative genomic hybridization	
Relative arguments that favor separate tumors (to be considered together with clinical factors):	
Different pattern of biomarkers	
Absence of nodal or systemic metastases	
Relative arguments that favor a single tumor source (to be considered together with clinical factors):	
Matching appearance on comprehensive histologic assessment	
Same biomarker pattern	
Significant nodal or systemic metastases	

M-M, Martini and Melamed; ACCP, the American College of Chest Physicians; AJCC, the American Joint Committee on Cancer.

The NGS platform has been widely used in the clinical diagnosis of MPLC in recent years (6, 12–16, 32, 34, 35). One study (13) found that compared to traditional histology (M-M), NGS may be a more accurate diagnostic and prognostic tool. Several studies (12, 14, 15) have compared the diagnostic efficacy of NGS and CHA and have shown that comprehensive NGS can clearly differentiate clonal relationships between different lesions compared to standard histopathologic methods, which are adequate in most cases but have considerable limitations in identifying IPM. The limitations of NGS itself mainly lie in the implementation cost and the need for personnel in bioinformatics, data analysis, and other fields, and it requires a longer time than pathological examinations. The diagnosis of histological evaluation remains necessary until NGS results are available. Comprehensive NGS can be used as complementary information to histology to achieve a robust identification of MPLC. Therefore, researchers have proposed new algorithms that integrate histologic and molecular approaches to diagnosis, which can substantially improve the discrimination ability for MPLC and IPM, saved about 15.5-22% of MPLC from being misdiagnosed (12, 14, 15).

#### 2.2.2 Chromosome variation

Array comparative genomic hybridization (aCGH) can identify genomic copy number variations. The study (24) showed that two tumors in one patient had deletions and amplifications of chromosome arms at the same location, and closely matched allelic changes were found on eight separate chromosome arms, resulting in a diagnosis of IPM. The study released by the Mayo Clinicin 2014 detecting chromosomal rearrangements in multiple tumors using NGS showed shared chromosomal rearrangement events between primary tumors and metastases, while no shared breakpoints were found between different tumors (40). In the study published in 2019 (29), using mate pair sequencing (MPseq) NGS to detect somatic mutations, chromosome copy number variants, and chromosomal rearrangements in tumor samples from patients with multiple lung cancers, the results showed that there were a large number of shared chromosomal rearrangements and somatic mutations as well as overlapping chromosome copy number variants in both tumors from patients with IPM, with no associated chromosomal variants between primary tumors and a large number of unique somatic mutations in each.

#### 2.2.3 Whole genome sequencing/whole exon sequencing

WGS/WES can help researchers obtain comprehensive genomic information in different tumors and thus perform finer tumor clonal relationship analysis and evolutionary studies. Liu et al. conducted WES/WGS and microarray-based CGH on multiple lesions (15 adenocarcinoma specimens and 1 metastatic lymph node) in 6 patients with synchronous multiple lung cancer (18). The results showed that different tumors from the same patient had different genomic features, including somatic mutations, copy number mutations, and chromosomal structural variations. Approximately 26% of the mutations were shared between the primary lesions and the metastatic lymph nodes. In addition, the investigators discovered that the same cancer gene showed different mutations in different tumors of one patient (KRAS mutation in P1: T1 p.G12A, T3 p.G12V; EGFR mutation in P4: T1 p.L858R, T2 p.S229C). Maet al. conducted WES sequencing on multiple tumor samples from four treated patients with multifocal lung cancer, and all tumors were confirmed to be primary tumors by constructing a phylogenetic tree (43). They found biological convergence of heterogeneous driving events on the same signaling pathway between different foci in the same patient, suggesting that the evolutionary trajectories of independent tumors in sMPLC may converge, leading to preferential activation of restricted key oncogenic pathways.

Additionally, in various studies, scholars have attempted to use WES to distinguish genomic heterogeneity and clonal relationships in multiple tumors or multiple GGNs, and only Corsini’s study obtained significant results (44). Yang et al. failed to better distinguish between IPM and MPLC in their study (45), and Zhou failed to determine the genetic characteristics and clonal relationships of most multiple GGNs (46). Tian et al. tested samples of MPLC with lymph node metastasis (LNM) and found that the genes with the highest mutation frequencies in this pair were MUC16, FLNA, MUC4, FAT3, SETD2, and CALR, which implied that MPLC with LNM may have a unique mutation spectrum (47).

#### 2.2.4 Others

Yu’s study compared differences in genetic, epigenetic, and immunological landscapes between isolated lung cancer and sMPLC (48). The results showed that sMPLC lesions had relatively lower TMB levels than isolated lung cancer, indicating caution when applying immunotherapy to sMPLC patients. Moreover, DNA methylation patterns and associated immune profiles were significantly different between sMPLC and isolated lung cancer, suggesting that they play an important role in the initiation and development of sMPLC. Research showed that MPLC and IPM could be better differentiated according to the differential rates of expression of cancer-associated proteins (p53, p16, p27, and c-erbB2) in the lesions (7). Two groups of patients with MPLC and LNM were included in the study for comparison, and the analysis showed that when the total protein expression difference rate was high, it was MPLC, and when the protein expression difference rate was low, it was IPM. Patients categorized in this way showed a significant difference in the 5-year survival rate.

In conclusion, molecular analysis provides new ideas and methods for the fine diagnosis of MPLC patients. Currently, the most widely used method in clinical practice is large-scale cancer-related gene detection based on NGS panels. Researches on the immune landscape, DNA methylation, and WGS have broad prospects and is still in full swing. It is worth noting that molecular analysis should be used as a supplement to clinical and pathological diagnosis.

### 2.3 Imaging diagnosis

#### 2.3.1 Chest CT

In the 8^th^ TNM staging (2), multifocal lung cancer is classified into four types: SPLC, separate tumor node (IPM), multifocal ground glass/lepidic (GG/L) nodes, and pneumonic type of adenocarcinoma. SPLC and multifocal GG/L nodes can be considered sMPLC, which are characterized by “two or more distinct masses with imaging characteristics of lung cancer (e.g., spiculated)” and “multiple ground-glass or part-solid nodules.” Currently, patients with multiple lung adenocarcinomas are commonly seen in clinical practice. In multiple studies (49, 50), multiple lung adenocarcinoma was categorized into 3-4 types according to the proportion of solid and GGO components, such as pure ground-glass node (GGN), GGO-predominant part solid node (PSN), solid-predominant PSN, and pure solid node. Zhang et al. identified multiple solid tumors as an independent risk factor for reduced RFS [hazard ratio (HR) 2.941; 95% confidence interval (CI) 1.07-8.08; P = 0.036] and poor OS (HR 6.13; 95% C1 1.15-32.63; P = 0.034) in patients with multiple lung adenocarcinomas (50). In addition, CT imaging manifestations can assist in distinguishing pathologic subtypes (51), and patients presenting with GGO or mixed GGO are more likely to have EGFR mutations (52). In conclusion, the CT manifestations of SPLC and multiple GGOs or partial solid nodules are referred to as MPLC, and multiple solid tumors are more prone to have advanced disease, which should be diagnosed with caution.

#### 2.3.2 PET-CT

^18^F-Fluoro-D-glucose positron emission tomography/computed tomography (^18^F-FDG PET/CT) plays an important role in the diagnosis of MPLC and has good diagnostic efficacy in monitoring patients with non-small cell lung cancer for post-treatment recurrence or the development of second primary cancers (53). The most commonly used semiquantitative parameter indicator in PET/CT imaging is the standardized uptake value (SUV), which refers to the ratio of the activity of a unit volume of tissue uptake of a contrast medium to the average activity of the whole-body tissue. The maximum SUV (SUV_max_) is most used technique because of its better repeatability and is generally applied to identify benign and malignant tumors. Liu’s (54) research shows that there is a significant difference in the ratio of SUV_max_ between different lesions in sMPLC and IPM, which can be used to distinguish sMPLC and IPM. The Lv study proposed the use of the dynamic ^18^F-FDG PET/CT acquisition mode and Patlak model to fit K_i_, representing the 18F-FDG net uptake rate constant, as well as the corresponding maximum diameter (D_max_) of the two largest tumors (55). By determining the absolute difference between SUV_max_/D_max_ and K_i_/D_max_ tumors (Δ SUV_max_/D_max_; Δ K_i_/D_max_), it was found that there was no significant difference in ΔSUV_max_/D_max_ between the IPM group and the sMPLC group, while Δ K_i_/D_max_ in the IPM group was significantly higher than that in the sMPLC group.

In addition, Suh et al. combined CT imaging features with PET/CT and designed a four-step algorithm: CT lesion types (Step 1), CT lesion morphology (Step 2), difference in maximal standardized uptake values on PET/CT (Step 3), and presence of N2/3 lymph node metastasis or distant metastasis (Step 4) (56). The results showed significantly higher specificity and positive predictive value for the diagnosis of IPM in all observers compared to without using the algorithm. Combining comprehensive imaging information is more helpful in improving the accuracy of diagnosing MPLC.

Overall, imaging diagnosis is particularly important for clinicians, especially thoracic surgeons for determining the surgeon’s judgment of surgical strategy. Multiple GGOs or partial solid nodules are often MPLC, while multiple solid nodules are difficult to make a clear diagnosis. Cases that are difficult to diagnose definitively can be defined by combining PET-CT and CT, supplemented by other clinical information.

Unlike previous disease diagnoses, the diagnosis of MPLC is more of a “spot the difference,” where multiple lesions are compared for similarities to determine whether they are primary or metastatic. Comparative methods include histology, imaging, molecular biology, etc. Imaging diagnosis is the key first-hand information but does not directly confirm the diagnosis as a preoperative diagnosis. Standard histopathological diagnosis is sufficient in most cases but has obvious limitations in identifying IPM and can only be determined after surgical resection, which should serve as a supplement to clinical information. Molecular biology provides a new direction for the fine diagnosis of MPLC, and its limitations mainly lie in the implementation cost and the need for personnel, such as specialists in bioinformatics and data analysis. It takes a longer time than pathologic examination. Therefore, diagnosis by histologic evaluation remains necessary until molecular diagnostic results are available. The guidelines emphasize that ideally, a multidisciplinary diagnosis should be made by combining all available information, including histopathology, imaging, molecular, and clinical features (9).

Throughout the development of diagnostic criteria for MPLC, the diagnosis of MPLC has become increasingly accurate, from the beginning of the process, which was based on the experience of clinicians only, to the idea of comprehensive diagnosis proposed in the current guidelines. Currently, there is no “one-size-fits-all” diagnostic gold standard that can be applied to all MPLC patients, so we should synthesize all the information that can be collected during the diagnostic process rather than using a single criterion to define MPLC patients.

## 3 Management

The management of MPLC mainly includes surgery, radiotherapy, and ablation, with targeted therapy and immunotherapy limited to specific patients (57–59). The quantity, location, and size of lesions in MPLC can affect the decision of its management. The NCCN guidelines state that a comprehensive multidisciplinary evaluation should be conducted, including thoracic radiology, pulmonary medicine, thoracic surgery, oncology, and radiation oncology. When the lesion is stable or grows very slowly, regular LDCT follow-up can be performed. If there is evidence of growth in the major nodule, surgery, radiotherapy or image-guided thermal ablation (IGTA) may be an option when there is a possibility of local radical treatment. If there is no possibility of local cure, the disease is treated as recurrent and metastatic.

### 3.1 Surgical treatment

Surgery is currently the treatment of choice for MPLC (60). The ACCP 3^rd^ guideline (8) recommends a multidisciplinary evaluation of patients with MPLC to exclude mediastinal and systemic metastases and to resect as many lesions as possible if the patient’s lung function permits. PET or PET-CT scanning is recommended for staging and to detect unsuspected metastatic disease (61). In case of CT-enlarged or PET-positive mediastinal lymph nodes require tissue confirmation, tissue confirmation is indicated. Endosonography [endobronchial ultrasonography (EBUS)/esophageal ultrasonography (EUS)] with fine-needle aspiration (FNA) is the first choice (when available). If negative, surgical staging will be performed (62). The NCCN guidelines (version 3.2023) recommend that anatomic pneumonectomy with parenchymal preservation is preferred in most patients undergoing surgery, especially in patients with insufficient lung function or other comorbidities that preclude lobectomy.

Researchers in many previous studies (60, 63–70) have discussed the choice between lobectomy and sublobar resection for the extent of surgical resection, with conflicting conclusions (**Table 2**). The reasons for the differences in these research conclusions may be due to differences in diagnostic criteria, characteristics of the included patients, and sample size, all of which were derived from retrospective studies. In Yang’s study, propensity score matching was utilized to reduce the potential bias caused by differences in the clinicopathological characteristics of first and second lung cancers, and the results showed that for tumors up to 2 cm in diameter, the prognosis of sublobar resection was comparable to that of lobectomy, and the survival rate of patients who underwent lobectomy for tumors between 2-3 cm was significantly higher than that of patients who underwent sublobar resection (P < 0.014) (71). In Niu’s study, patients with sMPLC were divided into sublobar resection and lobectomy groups and compared with single primary lung cancer. the results showed that there was no significant difference in 5-year OS (86.7% vs. 83.9%, P=0.482) and DFS (86.7% vs. 83.9%, P=0.482) between the lobectomy and sublobar resection groups, and that the sublobar resection group had a lower postoperative complication incidence and shorter postoperative hospitalization (72).

**Table 2:** Classification of lung cancers with multiple pulmonary sites of involvement in the eighth edition of the TNM (2).

Variable	Second Primary Lung Cancer	Separate tumor nodule (IPM)	Multifocal GG/L nodules	Pneumonic type of adenocarcinoma
Imaging features	Two or more distinct masses with imaging characteristics of lung cancer (eg, spiculated)	Typical lung cancer (eg, solid, spiculated) with separate solid nodule	Multiple ground-glass or part-solid nodules	Patchy areas of ground glass and consolidation
Pathologic features	Different histotype or different morphology by comprehensive histologic assessment	Distinct masses with the same morphologic features by comprehensive histologic assessment	Adenocarcinomas with prominent lepidic component (typically varying degrees of AIS, MIA, LPA)	Same histologic features throughout (most often invasive mucinous adenocarcinoma)
TNM classification	Separate cTNM and pTNM for each cancer	Location of separate nodule relative to primary site determines if T3, T4 or M1a; single N and M	T based on highest T lesion with (#/m) indicating multiplicity; single N and M	T based on size or T3 if in single lobe, T4 or M1a if in different ipsilateral or contralateral lobes; single N and M
Conceptual view	Unrelated tumors	Single tumor, with intrapulmonary metastasis	Separate tumors, albeit with similarities	Single tumor, diffuse pulmonary involvement

IPM, intrapulmonary metastasis; AIS, adenocarcinoma in situ; GG/L, ground glass/lepidic; LPA, lepidic-predominant adenocarcinoma; MIA, minimally invasive adenocarcinoma; p, pathologic; TNM, tumor, node, metastasis.

For patients with bMPLC, it is necessary to consider performing one-stage or two-stage operations. In Xu’s study, patients with bMPLC were divided into one-stage group and two-stage group, resulting in shorter operative time, surgical bleeding, pleural drainage, chest tube duration, and postoperative admission duration in the stage 1 group (P < 0.05) (73). It can be seen that in patients with bMPLC, simultaneous surgery may have better results when the patient’s cardiopulmonary function permits. In addition, many studies (64, 74–77) believe that surgical intervention for second primary lung cancer is effective. Hamaji’s study included patients with mMPLC who underwent secondary surgery after complete resection (78). The results showed that the 5-year OS rates after the first and second surgery were 87.4% and 60.8%, respectively. Both univariate and multivariate analyses showed that a lower-than-expected percentage of FEV1 and an ipsilateral secondary surgery were associated with an increased risk of postoperative complications. Kang’s study reviewed patients with sMPLC and mMPLC who received multiple pulmonary resection (MPR) and showed that the 5-year disease-free survival rate in the metachronous group was 85.4%, while it was 69.1% in the synchronous group; the 5-year overall survival rates were 86.1% and 84.8%, respectively, indicating that MPR is feasible for MPLC patients (64). Fourdrain et al. proposed that for mMPLC and bilateral sMPLC, long-term survival after secondary surgery is acceptable (3-year survival was 77%), and minimally invasive sublobar resection should be applied as much as possible (75).

Hattori et al. found that the presence of a GGO component in MPLC could differentiate survival, with a significant difference in 5-year OS between all lesions with a GGO component and all with a solid component (97.2% vs. 41.3%, P < 0.001), and multiple GGO foci and the absence of lymph node involvement were independent predictors of a good prognosis (79). Therefore, as surgical treatment of MPLC, anatomical resection with preservation of the lung parenchyma as much as possible should be opted for, and the extent of resection should be determined according to the size and location of the tumor. The necessity of surgical intervention should be evaluated for patients with multiple GGOs.

In summary, for MPLC that can be surgically removed, sublobar resection should be preferred based on the location and size of the lesions. For mMPLC and bilateral sMPLC, multiple pulmonary resection can be performed when the patient’s lung function permits. Multiple GGOs have a better prognosis and should be evaluated for the need for surgical intervention.

### 3.2 Radiotherapy

Stereotactic body radiation therapy (SBRT) has become the standard alternative treatment for patients with early-stage inoperable lung cancer (80, 81). It has limited toxicity and achieves satisfactory local control of tumors (82, 83). It has been widely used in MPLC (84), especially in patients with mMPLC (85). The American Society for Radiation Oncology (ASTRO)/American Society of Clinical Oncology (ASCO) guidelines (86, 87) strongly recommend SBRT as a potential curative treatment option for mMPLC patients, including those who underwent pneumonectomy (88). MPLC patients should be evaluated by a multidisciplinary team, and sMPLC patients may also be treated with SBRT as a therapeutic option when available. Research has shown that the PFS, OS, and incidence of local failure of sMPLC patients treated with SBRT are comparable to those in the cohort of patients with isolated early NSCLC (89). In another study, SBRT was found to have limited toxicity for the treatment of sMPLC, but the rate of progression appeared to be relatively high, with 11 patients (61%) experiencing disease progression (90). In iSABR trial, freedom from local recurrence at 1 year was 94% (90%CI, 87%-97%) for group MPLC (91). The RTOG 0618 trial (92) demonstrated that a high rate of intrathoracic control (96%) was achieved with the application of SBRT in patients with early-stage resectable lung cancer, but the guidelines (86) emphasized that surgery is still the recommended treatment for early resectable NSCLC patients and that SBRT should be considered as a potential alternative to surgery for patients at high risk of surgery. SBRT may reduce the risk of treatment in the short term, but the long-term outcome is uncertain over 3 years. Overall, SBRT can be adopted as an alternative treatment option for inoperable MPLC.

### 3.3 Image guided thermal ablation

IGTA is an option for select patients not receiving SABR or radical radiotherapy. Study has shown that IGTA provides at most temporary reductions in FEV1 and DLCO, with no statistically significant difference from baseline after healing (93, 94). For patients with high surgical risk (inoperable due to comorbidities), IGTA may be considered after a multidisciplinary evaluation. Ablative therapy may be associated with higher rates of local recurrence and complications when the lesion diameter is over 3 cm (95). Overall, patients with MPLC benefit from a comprehensive multidisciplinary approach combining surgery, SBRT, and IGTA to balance local control and preservation of lung function.

### 3.4 Targeted therapy

Targeted therapy is mostly used to treat patients with advanced NSCLC and is a potential alternative treatment option for selected MPLC patients whose lesions are not completely eradicated by surgery. YE et al. reported a strategy of continuing treatment of gefitinib-sensitive multiple GGO lesions with gefitinib after surgical resection of gefitinib-insensitive lesions, which eventually led to complete remission in this patient (96). Cheng et al. reported a case of an sMPLC patient who underwent surgical resection of the main lesion with EGFR mutation and received EGFR-TKI treatment for 3 months (97). The largest residual GGO lesion achieved complete remission, while the remaining lesions shrank or remained stable. Combination targeted therapy may be considered when there are different driver mutations between multiple lesions in MPLC. Aredo et al. reported a case of MPLC with different driving mutations in EGFR/RET treated with a combination of osimertinib and alectinib (98), and Chen et al. reported another patient with multiple primary lung adenocarcinoma who simultaneously had CD74-NRG1 fusion, EGFR, and human epidermal growth factor receptor 2 (ERBB2) mutations (99). The patient received a combination of afatinib and pyrotinib and showed a significant therapeutic response. Surgery combined with targeted therapy or targeted drug combination therapy for MPLC is promising, but it is necessary to further clarify the dosage and safety of targeted drugs.

### 3.5 Immunotherapy

Immunotherapy has brought cancer treatment into a new era, especially in advanced NSCLC without driver mutations. MPLC typically exhibits low TMB, low programmed cell death-ligand 1 (PD-L1), and heterogeneous immune infiltration landscapes (100, 101). A total of 13.4% of lesions in MPLC showed positive PD-L1 expression, while 27.9% (12/43) of patients showed inconsistent PD-L1 expression (102). The incidence of PD-L1-negative tumors in MPLC was higher in women and patients who had never smoked or were light smokers (103), and there was a higher incidence of PD-L1-positive tumors in wild-type tumors. This shows that PD-L1 expression levels are heterogeneous in MPLC (4), and the use of neoadjuvant programmed cell death-1 (PD-1)/PD-L1 inhibitors alone may not be the optimal treatment strategy for MPLC.

Lin et al. reported a case of an mMPLC patient with high PD-L1 expression detected after resection of a primary lung adenocarcinoma lesion; neoplastic tumors were found after a period of treatment with pembrolizumab and were later confirmed to be a PD-L1-negative small cell lung cancer (104). The patient was later treated with atezolizumab and achieved a durable clinical benefit. WES and immunohistochemistry confirmed differences in the distribution of CD8+ and CD4+ T cells in the two tumor tissues. Wu et al. reported a case of a patient with sMPLC who received 3 cycles of pemetrexed plus pembrolizumab upfront and surgical resection of a nearly pure solid lesion, followed by no further treatment (5). After 12 months of follow-up subsequent to termination of immunotherapy, almost all remaining lesions achieved radiographic complete remission due to the trailing effect of the PD-1 antibody of pembrolizumab.

Xu et al. conducted a clinical trial (ChiCTR1900022159) involving treatment of residual GGO lesions with the PD-1 inhibitor sintilimab (105). Of 13 GGOs in 7 patients who underwent secondary surgical resection, major pathologic response (MPR) was achieved in 2 lesions (2/13, 15%). Partial pathologic response (pPR) was found in 6 nodules (6/13, 46.2%), of which 4 GGOs were pPR high (>10% and <50% viable cells) and 2 were pPR low (>50% and <90% viable cells) lesions. pNR was observed in 5 (5/13, 38.5%) GGOs. Immunofluorescence revealed enriched T and B cells associated with pathological tumor regression. Patients who achieved MPR had higher TMB before the application of immunotherapy and were enriched for mutations such as MUC19 and PCDHB5, which have been found to be associated with good immunotherapeutic outcome.

In conclusion, the combined application of immunotherapy has a promising future, especially for PD-L1-positive patients with multiple GGOs who underwent surgery on the main lesion. Immune checkpoint inhibitor (ICI) application is expected to achieve complete remission of the remaining lesions, and further validation of the approach in large-sample clinical trials is currently pending.

## 4 Conclusions and future perspectives

MPLC is now very common in clinical practice and is characterized by multiple GGOs or subsolid nodules. Clinical diagnosis mainly relies on histopathologic, molecular, and imaging methods. Diagnostic imaging is indispensable as a preoperative diagnosis, histopathologic methods are adequate in most cases, and molecular diagnosis can make up for the lack of histology in differentiating IPM. It is worth noting that there are no universal diagnostic criteria for MPLC, and clinicians should collect as much comprehensive information as possible to make a diagnosis.

Treatment of MPLC should be developed by a multidisciplinary team. For patients who are surgically curable and can tolerate surgery, anatomical lung resection is preferred, preserving as much lung parenchyma as possible. SBRT can be an alternative option for inoperable patients, with better local control and low toxicity. IGTA is an option for selected patients who do not undergo SABR. Targeted therapies and immunotherapy are not yet standard therapies for the treatment of MPLC but have a promising future. Overall, patients with MPLC benefit from combination therapy combining surgery, SBRT, IGTA, targeted therapy, and immunotherapy to balance local control and preservation of lung function.

Currently, there is still much room for improvement in the diagnosis and treatment of MPLC, and the establishment of authoritative and comprehensive guidelines is needed. The application of AI technology to assist diagnosis is expected to further improve the efficacy of diagnosis in imaging and histopathology. cDNA testing, single-cell sequencing, and spatial transcriptome sequencing, which are molecular tests applied to MPLC, may bring hope for the discovery of new biomarkers or therapeutic targets. Impressive progress has been made in the field of emerging tumor vaccines, such as RNA vaccines, drug delivery through nanorobots and the potential for future applicability to the treatment of MPLC is promising. Multidisciplinary intersections allow for more flexible treatment options for MPLC, benefiting more patients.

## References

[1] Martini N Melamed MR. Multiple primary lung cancers. J Thorac Cardiovasc Surg. (1975) 70:606–12. doi: 10.1016/S0022-5223(19)40289-4 170482

[2] Rami-Porta R Asamura H Travis WD Rusch VW. Lung cancer — major changes in the American Joint Committee on Cancer eighth edition cancer staging manual. Ca: A Cancer J Clin. (2017) 67:138–55. doi: 10.3322/caac.21390 28140453

[3] Shen KR Meyers BF Larner JM Jones DR. Special treatment issues in lung cancer. Chest. (2007) 132:290S–305S. doi: 10.1378/chest.07-1382 17873175

[4] Mansfield AS Murphy SJ Peikert T Yi ES Vasmatzis G Wigle DA. Heterogeneity of programmed cell death ligand 1 expression in multifocal lung cancer. Clin Cancer Res. (2016) 22:2177–82. doi: 10.1158/1078-0432.CCR-15-2246 PMC4854782 26667490

[5] Wu S Li D Chen J Chen W Ren F. Tailing effect of PD-1 antibody results in the eradication of unresectable multiple primary lung cancer presenting as ground-glass opacities: a case report. Ann Palliat Med. (2021) 10:778–84. doi: 10.21037/apm-20-2132 33545799

[6] Pei G Li M Min X Liu Q Li D Yang Y. Molecular identification and genetic characterization of early-stage multiple primary lung cancer by large-panel next-generation sequencing analysis. Front Oncol. (2021) 11:653988. doi: 10.3389/fonc.2021.653988 34109114 PMC8183821

[7] Ono K Sugio K Uramoto H Baba T Ichiki Y Takenoyama M. Discrimination of multiple primary lung cancers from intrapulmonary metastasis based on the expression of four cancer-related proteins. Cancer-Am Cancer Soc. (2009) 115:3489–500. doi: 10.1002/cncr.24382 19452548

[8] Kozower BD Larner JM Detterbeck FC Jones DR. Special treatment issues in non-small cell lung cancer. Chest. (2013) 143:e369S–99S. doi: 10.1378/chest.12-2362 23649447

[9] Detterbeck FC Franklin WA Nicholson AG Girard N Arenberg DA Travis WD. The IASLC lung cancer staging project: background data and proposed criteria to distinguish separate primary lung cancers from metastatic foci in patients with two lung tumors in the forthcoming eighth edition of the TNM classification for lung cancer. J Thorac Oncol. (2016) 11:651–65. doi: 10.1016/j.jtho.2016.01.025 26944304

[10] Arai J Tsuchiya T Oikawa M MoChinaga K Hayashi T Yoshiura K. Clinical and molecular analysis of synchronous double lung cancers. Lung Cancer. (2012) 77:281–7. doi: 10.1016/j.lungcan.2012.04.003 22560922

[11] Asmar R Sonett JR Singh G Mansukhani MM Borczuk AC. Use of oncogenic driver mutations in staging of multiple primary lung carcinomas: A single-center experience. J Thorac Oncol. (2017) 12:1524–35. doi: 10.1016/j.jtho.2017.06.012 28647671

[12] Chang JC Alex D Bott M Tan KS Seshan V Golden A. Comprehensive next-generation sequencing unambiguously distinguishes separate primary lung carcinomas from intrapulmonary metastases: comparison with standard histopathologic approach. Clin Cancer Res. (2019) 25:7113–25. doi: 10.1158/1078-0432.CCR-19-1700 PMC7713586 31471310

[13] Goodwin D Rathi V Conron M Wright GM. Genomic and clinical significance of multiple primary lung cancers as determined by next-generation sequencing. J Thorac Oncol. (2021) 16:1166–75. doi: 10.1016/j.jtho.2021.03.018 33845213

[14] Mansuet-Lupo A Barritault M Alifano M Janet-Vendroux A Zarmaev M Biton J. Proposal for a combined histomolecular algorithm to distinguish multiple primary adenocarcinomas from intrapulmonary metastasis in patients with multiple lung tumors. J Thorac Oncol. (2019) 14:844–56. doi: 10.1016/j.jtho.2019.01.017 30721797

[15] Zhang X Fan X Sun C Wang L Miao Y Wang L. A novel NGS-based diagnostic algorithm for classifying multifocal lung adenocarcinomas in pN0M0 patients. J Pathol: Clin Res. (2022) 9:108–20. doi: 10.1002/cjp2.306 PMC9896159 36579550

[16] Zheng R Shen Q Mardekian S Solomides C Wang Z Evans NR. Molecular profiling of key driver genes improves staging accuracy in multifocal non–small cell lung cancer. J Thorac Cardiovasc Surg. (2020) 160:e71–9. doi: 10.1016/j.jtcvs.2019.11.126 32007245

[17] Xie H Dou S Huang X Wen Y Yang L. The effect of spread through air spaces on postoperative recurrence-free survival in patients with multiple primary lung cancers. World J Surg Oncol. (2024) 22:75. doi: 10.1186/s12957-024-03351-3 38443963 PMC10913208

[18] Liu Y Zhang J Li L Yin G Zhang J Zheng S. Genomic heterogeneity of multiple synchronous lung cancer. Nat Commun. (2016) 7:13200. doi: 10.1038/ncomms13200 27767028 PMC5078731

[19] de Bruin EC McGranahan N Mitter R Salm M Wedge DC Yates L. Spatial and temporal diversity in genomic instability processes defines lung cancer evolution. Sci (New York N.Y.). (2014) 346:251–6. doi: 10.1126/science.1253462 PMC4636050 25301630

[20] Zhang J Fujimoto J Zhang J Wedge DC Song X Zhang J. Intra-tumor heterogeneity in localized lung adenocarcinomas delineated by multi-region sequencing. Sci (New York N.Y.). (2014) 346:256–9. doi: 10.1126/science.1256930 PMC4354858 25301631

[21] Shen C Xu H Liu L Zhou Y Chen D Du H. Unique trend” and “contradictory trend” in discrimination of primary synchronous lung cancer and metastatic lung cancer. BMC Cancer. (2013) 13:467. doi: 10.1186/1471-2407-13-467 24106770 PMC3852048

[22] Shen C Wang X Tian L Che G. Microsatellite alteration in multiple primary lung cancer. J Thorac Dis. (2014) 6:1499–505. doi: 10.3978/j.issn.2072-1439.2014.09.14 PMC4215141 25364529

[23] Chang Y Wu C Lin S Hsiao C Jou Y Lee Y. Clonality and prognostic implications of p53 and epidermal growth factor receptor somatic aberrations in multiple primary lung cancers. Clin Cancer Res. (2007) 13:52–8. doi: 10.1158/1078-0432.CCR-06-1743 17200338

[24] Girard N Ostrovnaya I Lau C Park B Ladanyi M Finley D. Genomic and mutational profiling to assess clonal relationships between multiple non–small cell lung cancers. Clin Cancer Res. (2009) 15:5184–90. doi: 10.1158/1078-0432.CCR-09-0594 PMC2892178 19671847

[25] Higuchi R Nakagomi T Goto T Hirotsu Y Shikata D Yokoyama Y. Identification of clonality through genomic profile analysis in multiple lung cancers. J Clin Med. (2020) 9:573. doi: 10.3390/jcm9020573 32093372 PMC7074554

[26] Iwata T Sugio K Uramoto H Yamada S Onitsuka T Nose N. Detection of EGFR and K-ras mutations for diagnosis of multiple lung adenocarcinomas. Front Biosci (Landmark Edition). (2011) 16:2961–9. doi: 10.2741/3891 21622214

[27] Matsuzoe D Hideshima T Ohshima K Kawahara K Shirakusa T Kimura A. Discrimination of double primary lung cancer from intrapulmonary metastasis by p53 gene mutation. Brit J Cancer. (1999) 79:1549–52. doi: 10.1038/sj.bjc.6690247 PMC2362717 10188905

[28] Mitsudomi T Yatabe Y Koshikawa T Hatooka S Shinoda M Suyama M. Mutations of the p53 tumor suppressor gene as clonal marker for multiple primary lung cancers. J Thorac Cardiovasc Surg. (1997) 114:354–60. doi: 10.1016/S0022-5223(97)70180-6 9305187

[29] Murphy SJ Harris FR Kosari F Barreto Siqueira Parrilha Nasir A Johnson SH. Using genomics to differentiate multiple primaries from metastatic lung cancer. J Thorac Oncol. (2019) 14:1567–82. doi: 10.1016/j.jtho.2019.05.008 31103780

[30] Noguchi M Maezawa N Nakanishi Y Matsuno Y Shimosato Y Hirohashi S. Application of the p53 gene mutation pattern for differential diagnosis of primary versus metastatic lung carcinomas. Diagn Mol Pathol: Am J Surg Pathol Part B. (1993) 2:29–35. doi: 10.1097/00019606-199303000-00005 8287223

[31] Pagan CA Shu CA Crapanzano JP Lagos GG Stoopler MB Rizvi NA. Synchronous pulmonary adenocarcinomas. Am J Clin Pathol. (2020) 154:57–69. doi: 10.1093/ajcp/aqaa023 32146481

[32] Saab J Zia H Mathew S Kluk M Narula N Fernandes H. Utility of genomic analysis in differentiating synchronous and metachronous lung adenocarcinomas from primary adenocarcinomas with intrapulmonary metastasis. Transl Oncol. (2017) 10:442–9. doi: 10.1016/j.tranon.2017.02.009 PMC5406583 28448960

[33] van Rens MTM Eijken EJE Elbers JRJ Lammers JJ Tilanus MGJ Slootweg PJ. p53 mutation analysis for definite diagnosis of multiple primary lung carcinoma. Cancer-Am Cancer Soc. (2002) 94:188–96. doi: 10.1002/cncr.10001 11815976

[34] Wang X Gong Y Yao J Chen Y Li Y Zeng Z. Establishment of criteria for molecular differential diagnosis of MPLC and IPM. Front Oncol. (2021) 10:614430. doi: 10.3389/fonc.2020.614430 33552986 PMC7860975

[35] Wang Y Wang G Zheng H Liu J Ma G Huang G. Distinct gene mutation profiles among multiple and single primary lung adenocarcinoma. Front Oncol. (2022) 12:1014997. doi: 10.3389/fonc.2022.1014997 36531058 PMC9755731

[36] Zhu D Cao D Shen M Lv J. Morphological and genetic heterogeneity of synchronous multifocal lung adenocarcinoma in a Chinese cohort. BMC Cancer. (2021) 21:176. doi: 10.1186/s12885-021-07892-8 33602172 PMC7890910

[37] Vincenten J van Essen HF Lissenberg-Witte BI Bulkmans N Krijgsman O Sie D. Clonality analysis of pulmonary tumors by genome-wide copy number profiling. PloS One. (2019) 14:e0223827. doi: 10.1371/journal.pone.0223827 31618260 PMC6795528

[38] Huang J Behrens C Wistuba I Gazdar AF Jagirdar J. Molecular analysis of synchronous and metachronous tumors of the lung: impact on management and prognosis. Ann Diagn Pathol. (2001) 5:321–9. doi: 10.1053/adpa.2001.29338 11745069

[39] Bonanno L Pavan A Indraccolo S. Assessment of chromosomal rearrangements helps to differentiate multiple lung primary cancers from metastases. Transl Lung Cancer R. (2019) 8:S435–8. doi: 10.21037/tlcr.2019.11.09 PMC6987365 32038932

[40] Murphy SJ Aubry M Harris FR Halling GC Johnson SH Terra S. Identification of independent primary tumors and intrapulmonary metastases using DNA rearrangements in non-small-cell lung cancer. J Clin Oncol. (2014) 32:4050–8. doi: 10.1200/JCO.2014.56.7644 PMC4879716 25385739

[41] Vokes NI Zhang J. The role of whole exome sequencing in distinguishing primary and secondary lung cancers. Lung Cancer: Targets Ther. (2021) 12:139–49. doi: 10.2147/LCTT.S272518 PMC8648100 34880699

[42] Mok TS Wu YL Thongprasert S Yang CH Chu DT Saijo N. Gefitinib or carboplatin-paclitaxel in pulmonary adenocarcinoma. New Engl J Med. (2009) 361:947–57. doi: 10.1056/NEJMoa0810699 19692680

[43] Ma P Fu Y Cai M Yan Y Jing Y Zhang S. Simultaneous evolutionary expansion and constraint of genomic heterogeneity in multifocal lung cancer. Nat Commun. (2017) 8:823. doi: 10.1038/s41467-017-00963-0 29018192 PMC5634994

[44] Corsini EM Wang J Wu C Fujimoto J Negrao MV Chen R. Genomic assessment distinguishes intrapulmonary metastases from synchronous primary lung cancers. J Thorac Dis. (2020) 12:1952–9. doi: 10.21037/jtd-20-1 PMC7330333 32642098

[45] Yang R Li P Wang D Wang L Yin J Yu B. Genetic and immune characteristics of multiple primary lung cancers and lung metastases. Thorac Cancer. (2021) 12:2544–50. doi: 10.1111/1759-7714.14134 PMC8487821 34510768

[46] Zhou D Liu Q Li M Hou B Yang G Lu X. Utility of whole exome sequencing analysis in differentiating intrapulmonary metastatic multiple ground-glass nodules (GGNs) from multiple primary GGNs. Int J Clin Oncol. (2022) 27:871–81. doi: 10.1007/s10147-022-02134-8 PMC9023437 35171361

[47] Tian H Wang Y Yang Z Chen P Xu J Tian Y. Genetic trajectory and clonal evolution of multiple primary lung cancer with lymph node metastasis. Cancer Gene Ther. (2023) 30:507–20. doi: 10.1038/s41417-022-00572-0 PMC10014582 36653483

[48] Yu F Huang X Zhou D Zhao Z Wu F Qian B. Genetic, DNA methylation, and immune profile discrepancies between early-stage single primary lung cancer and synchronous multiple primary lung cancer. Clin Epigenet. (2023) 15:4. doi: 10.1186/s13148-023-01422-y PMC9824942 36611170

[49] Huo J Luo T He X Gong J Lv F Li Q. Radiological classification, gene-mutation status, and surgical prognosis of synchronous multiple primary lung cancer. Eur Radiol. (2022) 32:4264–74. doi: 10.1007/s00330-021-08464-x 34989846

[50] Zhang Y Li G Li Y Liu Q Yu Y Ma Y. Imaging features suggestive of multiple primary lung adenocarcinomas. Ann Surg Oncol. (2020) 27:2061–70. doi: 10.1245/s10434-019-08109-w 31863415

[51] Ko JP Suh J Ibidapo O Escalon JG Li J Pass H. Lung adenocarcinoma: correlation of quantitative CT findings with pathologic findings. Radiology. (2016) 280:931–9. doi: 10.1148/radiol.2016142975 27097236

[52] Han X Fan J Gu J Li Y Yang M Liu T. CT features associated with EGFR mutations and ALK positivity in patients with multiple primary lung adenocarcinomas. Cancer Imaging. (2020) 20:51. doi: 10.1186/s40644-020-00330-1 32690092 PMC7372851

[53] Lim CH Park SB Kim HK Choi YS Kim J Ahn YC. Clinical value of surveillance 18F-fluorodeoxyglucose PET/CT for detecting unsuspected recurrence or second primary cancer in non-small cell lung cancer after curative therapy. Cancers. (2022) 14. doi: 10.3390/cancers14030632 PMC8833387 35158900

[54] Liu Y Tang Y Xue Z Jin X Ma G Zhao P. SUVmax ratio on PET/CT may differentiate between lung metastases and synchronous multiple primary lung cancer. Acad Radiol. (2020) 27:618–23. doi: 10.1016/j.acra.2019.07.001 31787567

[55] Lv W Yang M Zhong H Wang X Yang S Bi L. Application of dynamic 18F-FDG PET/CT for distinguishing intrapulmonary metastases from synchronous multiple primary lung cancer. Mol Imaging. (2022) 2022:8081299. doi: 10.1155/2022/8081299 35903246 PMC9281433

[56] Suh YJ Lee H Sung P Yoen H Kim S Han S. A novel algorithm to differentiate between multiple primary lung cancers and intrapulmonary metastasis in multiple lung cancers with multiple pulmonary sites of involvement. J Thorac Oncol. (2020) 15:203–15. doi: 10.1016/j.jtho.2019.09.221 31634666

[57] Chiang C Tsai P Yeh Y Wu Y Hsu H Chen Y. Recent advances in the diagnosis and management of multiple primary lung cancer. Cancers. (2022) 14:242. doi: 10.3390/cancers14010242 35008406 PMC8750235

[58] Chen C Huang X Peng M Liu W Yu F Wang X. Multiple primary lung cancer: a rising challenge. J Thorac Dis. (2019) 11:S523–36. doi: 10.21037/jtd.2019.01.56 PMC6465434 31032071

[59] Zhao L Liu C Xie G Wu F Hu C. Multiple primary lung cancers: A new challenge in the era of precision medicine. Cancer Manag Res. (2020) 12:10361–74. doi: 10.2147/CMAR.S268081 PMC7585808 33116891

[60] Jung EJ Lee JH Jeon K Koh W Suh GY Chung MP. Treatment outcomes for patients with synchronous multiple primary non-small cell lung cancer. Lung Cancer. (2011) 73:237–42. doi: 10.1016/j.lungcan.2010.11.008 21145616

[61] Silvestri GA Gonzalez AV Jantz MA Margolis ML Gould MK Tanoue LT. Methods for staging non-small cell lung cancer: Diagnosis and management of lung cancer, 3rd ed: American College of Chest Physicians evidence-based clinical practice guidelines. Chest. (2013) 143:e211S–50S. doi: 10.1378/chest.12-2355 23649440

[62] De Leyn P Dooms C Kuzdzal J Lardinois D Passlick B Rami-Porta R. Revised ESTS guidelines for preoperative mediastinal lymph node staging for non-small-cell lung cancer. Eur J Cardio-Thorac. (2014) 45:787–98. doi: 10.1093/ejcts/ezu028 24578407

[63] Ishikawa Y Nakayama H Ito H Yokose T Tsuboi M Nishii T. Surgical treatment for synchronous primary lung adenocarcinomas. Ann Thorac Surg. (2014) 98:1983–8. doi: 10.1016/j.athoracsur.2014.07.006 25443005

[64] Kang X Zhang C Zhou H Zhang J Yan W Zhong W. Multiple pulmonary resections for synchronous and metachronous lung cancer at two Chinese centers. Ann Thorac Surg. (2020) 109:856–63. doi: 10.1016/j.athoracsur.2019.09.088 31765616

[65] Kocaturk CI Gunluoglu MZ Cansever L Demir A Cinar U Dincer SI. Survival and prognostic factors in surgically resected synchronous multiple primary lung cancers. Eur J Cardio-Thoracic Surg. (2011) 39:160–6. doi: 10.1016/j.ejcts.2010.05.037 20650645

[66] Watanabe T Tanahashi M Suzuki E Yoshii N Tsuchida H Yobita S. Surgical treatment for synchronous multiple primary lung cancer: Is it possible to achieve both curability and preservation of the pulmonary function? Thorac Cancer. (2021) 12:2996–3004. doi: 10.1111/1759-7714.14164 34590424 PMC8590900

[67] Xiao F Liu D Guo Y Shi B Song Z Tian Y. Survival rate and prognostic factors of surgically resected clinically synchronous multiple primary non-small cell lung cancer and further differentiation from intrapulmonary metastasis. J Thorac Dis. (2017) 9:990–1001. doi: 10.21037/jtd.2017.03.59 28523154 PMC5418261

[68] Yang H Sun Y Yao F Yu K Gu H Han B. Surgical therapy for bilateral multiple primary lung cancer. Ann Thorac Surg. (2016) 101:1145–52. doi: 10.1016/j.athoracsur.2015.09.028 26602007

[69] Yu Y Hsu P Yeh Y Huang C Hsieh C Chou T. Surgical results of synchronous multiple primary lung cancers: similar to the stage-matched solitary primary lung cancers? Ann Thorac Surg. (2013) 96:1966–74. doi: 10.1016/j.athoracsur.2013.04.142 24021769

[70] Zuin A Andriolo LG Marulli G Schiavon M Nicotra S Calabrese F. Is lobectomy really more effective than sublobar resection in the surgical treatment of second primary lung cancer? Eur J Cardio-Thoracic Surg. (2013) 44:e120–5, discussion e125. doi: 10.1093/ejcts/ezt219 23657547

[71] Yang X Zhan C Li M Huang Y Zhao M Yang X. Lobectomy versus sublobectomy in metachronous second primary lung cancer: A propensity score study. Ann Thorac Surg. (2018) 106:880–7. doi: 10.1016/j.athoracsur.2018.04.071 29852145

[72] Niu N Zhou L Zhao J Ma X Yang F Qi W. Sublobar resection versus lobectomy in the treatment of synchronous multiple primary lung cancer. World J Surg Oncol. (2023) 21:135. doi: 10.1186/s12957-023-02996-w 37088839 PMC10124016

[73] Xu G Wang G Mei X Wu M Li T Xie M. Sequential pulmonary resections by uniportal video-assisted thoracic surgery for bilateral multiple pulmonary nodules. Front Oncol. (2022) 12:961812. doi: 10.3389/fonc.2022.961812 36263215 PMC9574321

[74] Abid W Seguin-Givelet A Brian E Grigoroiu M Girard P Girard N. Second pulmonary resection for a second primary lung cancer: analysis of morbidity and survival. Eur J Cardio-Thorac. (2021) 59:1287–94. doi: 10.1093/ejcts/ezaa438 33367556

[75] Fourdrain A Bagan P Georges O Lafitte S De Dominicis F Meynier J. Outcomes after contralateral anatomic surgical resection in multiple lung cancer. Thorac Cardiovasc Surgeon. (2021) 69:373–9. doi: 10.1055/s-0040-1710068 32443159

[76] Sato S Nakamura M Shimizu Y Goto T Kitahara A Koike T. Impact of postoperative complications on outcomes of second surgery for second primary lung cancer. Surg Today. (2020) 50:1452–60. doi: 10.1007/s00595-020-02038-y 32488477

[77] Zhou H Kang X Dai L Yan W Yang Y Lin Y. Efficacy of repeated surgery is superior to that of non-surgery for recurrent/second primary lung cancer after initial operation for primary lung cancer. Thorac Cancer. (2018) 9:1062–8. doi: 10.1111/1759-7714.12790 PMC6068452 29917320

[78] Hamaji M Allen MS Cassivi SD Deschamps C Nichols FC Wigle DA. Surgical treatment of metachronous second primary lung cancer after complete resection of non–small cell lung cancer. J Thorac Cardiovasc Surg. (2013) 145:683–91. doi: 10.1016/j.jtcvs.2012.12.051 23414986

[79] Hattori A Takamochi K Oh S Suzuki K. Prognostic classification of multiple primary lung cancers based on a ground-glass opacity component. Ann Thorac Surg. (2020) 109:420–7. doi: 10.1016/j.athoracsur.2019.09.008 31593656

[80] Matthiesen C Thompson JS de la Fuente Herman T Ahmad S Herman T. Use of stereotactic body radiation therapy for medically inoperable multiple primary lung cancer. J Med Imag Radiat On. (2012) 56:561–6. doi: 10.1111/j.1754-9485.2012.02393.x 23043577

[81] Timmerman R Paulus R Galvin J Michalski J Straube W Bradley J. Stereotactic body radiation therapy for inoperable early stage lung cancer. Jama-J Am Med Assoc. (2010) 303:1070–6. doi: 10.1001/jama.2010.261 PMC2907644 20233825

[82] Ricardi U Badellino S Filippi AR. Stereotactic body radiotherapy for early stage lung cancer: History and updated role. Lung Cancer (Amsterdam Netherlands). (2015) 90:388–96. doi: 10.1016/j.lungcan.2015.10.016 26791797

[83] Ricardi U Badellino S Filippi AR. What do radiation oncologists require for future advancements in lung SBRT? Physica Med. (2017) 44:150–6. doi: 10.1016/j.ejmp.2016.11.114 27914779

[84] Chang JY Liu Y Zhu Z Welsh JW Gomez DR Komaki R. Stereotactic ablative radiotherapy: a potentially curable approach to early stage multiple primary lung cancer. Cancer-Am Cancer Soc. (2013) 119:3402–10. doi: 10.1002/cncr.28217 PMC3775964 23798353

[85] Testolin A Favretto MS Cora S Cavedon C. Stereotactic body radiation therapy for a new lung cancer arising after pneumonectomy: dosimetric evaluation and pulmonary toxicity. Br J Radiol. (2015) 88. doi: 10.1259/bjr.20150228 PMC4743449 26290398

[86] Schneider BJ Daly ME Kennedy EB Antonoff MB Broderick S Feldman J. Stereotactic body radiotherapy for early-stage non–small-cell lung cancer: American society of clinical oncology endorsement of the american society for radiation oncology evidence-based guideline. J Clin Oncol. (2018) 36:710–9. doi: 10.1200/JCO.2017.74.9671 29106810

[87] Videtic GMM Donington J Giuliani M Heinzerling J Karas TZ Kelsey CR. Stereotactic body radiation therapy for early-stage non-small cell lung cancer: Executive Summary of an ASTRO Evidence-Based Guideline. Pract Radiat Oncol. (2017) 7:295–301. doi: 10.1016/j.prro.2017.04.014 28596092

[88] Dong B Chen R Zhu X Wu Q Jin J Wang W. Comparison of stereotactic body radiation therapy versus surgery for multiple primary lung cancers after prior radical resection: A multicenter retrospective study. Clin Transl Rad Onco. (2023) 40. doi: 10.1016/j.ctro.2023.100601 PMC10020093 36936471

[89] Steber CR Hughes RT Soike MH Helis CA Nieto K Jacobson T. Stereotactic body radiotherapy for synchronous early stage non-small cell lung cancer. Acta Oncol. (2021) 60:605–12. doi: 10.1080/0284186X.2021.1892182 PMC8996167 33645424

[90] Shintani T Masago K Takayama K Ueki K Kimino G Ueki N. Stereotactic body radiotherapy for synchronous primary lung cancer: clinical outcome of 18 cases. Clin Lung Cancer. (2015) 16:e91–6. doi: 10.1016/j.cllc.2014.12.011 25659504

[91] Gensheimer MF Gee H Shirato H Taguchi H Snyder JM Chin AL. Individualized stereotactic ablative radiotherapy for lung tumors: the iSABR phase 2 nonrandomized controlled trial. JAMA Oncol. (2023) 9:1525–34. doi: 10.1001/jamaoncol.2023.3495 PMC10502697 37707820

[92] Timmerman RD Paulus R Pass HI Gore EM Edelman MJ Galvin J. Stereotactic body radiation therapy for operable early-stage lung cancer: findings from the NRG oncology RTOG 0618 trial. JAMA Oncol. (2018) 4:1263. doi: 10.1001/jamaoncol.2018.1251 29852037 PMC6117102

[93] Lencioni R Crocetti L Cioni R Suh R Glenn D Regge D. Response to radiofrequency ablation of pulmonary tumours: a prospective, intention-to-treat, multicentre clinical trial (the RAPTURE study). Lancet Oncol. (2008) 9:621–8. doi: 10.1016/S1470-2045(08)70155-4 18565793

[94] Tada A Hiraki T Iguchi T Gobara H Mimura H Toyooka S. Influence of radiofrequency ablation of lung cancer on pulmonary function. Cardiovasc Inter Rad. (2012) 35:860–7. doi: 10.1007/s00270-011-0221-z 21725672

[95] Murphy MC Wrobel MM Fisher DA Cahalane AM Fintelmann FJ. Update on image-guided thermal lung ablation: society guidelines, therapeutic alternatives, and postablation imaging findings. Am J Roentgenol. (2022) 219:471–85. doi: 10.2214/AJR.21.27099 35319908

[96] Ye C Wang J Li W Chai Y. Novel strategy for synchronous multiple primary lung cancer displaying unique molecular profiles. Ann Thorac Surg. (2016) 101:e45–7. doi: 10.1016/j.athoracsur.2015.06.042 26777970

[97] Cheng B Deng H Zhao Y Zhu F Liang H Li C. Management for residual ground-glass opacity lesions after resection of main tumor in multifocal lung cancer: A case report and literature review. Cancer Manag Res. (2021) 13:977–85. doi: 10.2147/CMAR.S290830 PMC7868271 33568943

[98] Aredo JV Diehn M Berry GJ Wakelee HA. Targeted treatment of multiple primary lung cancers harboring distinct EGFR or RET alterations: A case report. Clin Lung Cancer. (2021) 22:e673–7. doi: 10.1016/j.cllc.2020.12.005 33451914

[99] Chen K Li W Xi X Zhong J. A case of multiple primary lung adenocarcinoma with a CD74-NRG1 fusion protein and HER2 mutation benefit from combined target therapy. Thorac Cancer. (2022) 13:3063–7. doi: 10.1111/1759-7714.14636 PMC9626339 36096509

[100] Hu C Zhao L Liu W Fan S Liu J Liu Y. Genomic profiles and their associations with TMB, PD-L1 expression, and immune cell infiltration landscapes in synchronous multiple primary lung cancers. J Immunother Cancer. (2021) 9:e003773. doi: 10.1136/jitc-2021-003773 34887263 PMC8663088

[101] Zhang C Yin K Liu S Yan L Su J Wu Y. Multiomics analysis reveals a distinct response mechanism in multiple primary lung adenocarcinoma after neoadjuvant immunotherapy. J Immunother Cancer. (2021) 9:e002312. doi: 10.1136/jitc-2020-002312 33820821 PMC8025811

[102] Haratake N Toyokawa G Takada K Kozuma Y Matsubara T Takamori S. Programmed death-ligand 1 expression and EGFR mutations in multifocal lung cancer. Ann Thorac Surg. (2018) 105:448–54. doi: 10.1016/j.athoracsur.2017.09.025 29254651

[103] Izumi M Sawa K Oyanagi J Noura I Fukui M Ogawa K. Tumor microenvironment disparity in multiple primary lung cancers: Impact of non-intrinsic factors, histological subtypes, and genetic aberrations. Transl Oncol. (2021) 14:101102. doi: 10.1016/j.tranon.2021.101102 33930847 PMC8102176

[104] Lin X Qiu G Li F Deng H Qin Y Xie X. Case report: A rare case of metachronous multiple primary lung cancers in a patient with successful management by switching from anti-PD-1 therapy to anti-PD-L1 therapy. Front Immunol. (2021) 12:683202. doi: 10.3389/fimmu.2021.683202 34149722 PMC8207139

[105] Xu L Shi M Wang S Li M Yin W Zhang J. Immunotherapy for bilateral multiple ground glass opacities: An exploratory study for synchronous multiple primary lung cancer. Front Immunol. (2022) 13:1009621. doi: 10.3389/fimmu.2022.1009621 36389707 PMC9642914

